# In Silico Analysis Identifies Differently Expressed lncRNAs as Novel Biomarkers for the Prognosis of Thyroid Cancer

**DOI:** 10.1155/2020/3651051

**Published:** 2020-04-23

**Authors:** Yuansheng Rao, Haiying Liu, Xiaojuan Yan, Jianhong Wang

**Affiliations:** Department of Otorhinolaryngology, Beijing Anzhen Hospital, No. 2 Anzhen Road, Chaoyang District, Beijing 100029, China

## Abstract

**Background:**

Thyroid cancer (TC) is one of the most common type of endocrine tumors. Long noncoding RNAs had been demonstrated to play key roles in TC. *Material and Methods*. The lncRNA expression data were downloaded from Co-lncRNA database. The raw data was normalized using the limma package in R software version 3.3.0. The differentially expressed mRNA and lncRNAs were identified by the linear models for the microarray analysis (Limma) method. The DEGs were obtained with thresholds of ∣logFC∣ > 1.5 and *P* < 0.001. The hierarchical cluster analysis of differentially expressed mRNAs and lncRNAs was performed using CLUSTER 3.0, and the hierarchical clustering heat map was visualized by Tree View.

**Results:**

In the present study, we identified 6 upregulated and 85 downregulated lncRNAs in TC samples. Moreover, we for the first time identified 16 downregulated lncRNAs was correlated to longer disease-free survival time in patients with TC, including ATP1A1-AS1, CATIP-AS1, FAM13A-AS1, LINC00641, LINC00924, MIR22HG, NDUFA6-AS1, RP11-175K6.1, RP11-727A23.5, RP11-774O3.3, RP13-895J2.2, SDCBP2-AS1, SLC26A4-AS1, SNHG15, SRP14-AS1, and ZNF674-AS1.

**Conclusions:**

Bioinformatics analysis revealed these lncRNAs were involved in regulating the RNA metabolic process, cell migration, organelle assembly, tRNA modification, and hormone levels. This study will provide useful information to explore the potential candidate biomarkers for diagnosis, prognosis, and drug targets for TC.

## 1. Introduction

Thyroid cancer (TC) is one of the most common type of endocrine tumors [1]. A recent study showed the incidence of TC increased rapidly worldwide, especially in female. However, there was still lacking of effective biomarkers for the prognosis of TC. Over the past decades, several genes were identified to be related to the progression of TC and could serve as potential biomarkers for TC, such as RAS [2] and BRAF (V600E) [3] gene mutations. Moreover, with the development of the next-generation sequencing method, a series of public datasets were developed to explore the potential biomarkers and mechanisms underlying tumor progression in human cancers. For example, Wang et al. analyzed TCGA dataset and found lncRNA UNC5B-AS1 promoted TC growth and metastasis [4]. Identification of novel biomarkers is still an urgent need for the TC.

Long noncoding RNAs (lncRNAs) were reported to play important roles in tumorigenesis and cancer progression [5]. LncRNAs bound to chromatin, proteins, and RNAs to modulate cancer proliferation, apoptosis, autophagy, epithelial-mesenchymal transition (EMT), and metastasis [6]. In TC, ENST00000539653 promoted cancer progression via MAPK signaling. TUG1 regulated TC cell proliferation and EMT through targeting miR-145 [7]. A recent study showed antisense lncRNA COMET repression inhibited cell viability and invasiveness and induced sensitivity to vemurafenib in BRAF- and RET-driven TC [8]. Interestingly, emerging studies demonstrated lncRNAs could serve as potential prognostic or diagnostic biomarkers for human cancers. For instance, Zhang et al. reported that downregulation of DANCR is a biomarker for TC diagnosis [9]. Decreased EMX2OS expression was associated with unfavorable recurrence-free survival (RFS) in classical PTC [10].

In this study, we identified differently expressed lncRNAs using two public datasets, including Co-lncRNA database and GEPIA database [11]. Then, coexpression network analysis, gene ontology (GO) analysis, and Kyoto Encyclopedia of Genes and Genomes (KEGG) pathway analysis were used to evaluate the potential functions of these lncRNAs in TC. We thought this study could provide novel biomarkers for TC.

## 2. Materials and Methods

### 2.1. Public Dataset Analysis

The lncRNA expression data were downloaded from Co-lncRNA database. Co-lncRNA database included 12 normal samples and 83 TC samples. The raw data was normalized using the limma package in R software version 3.3.0 (https://www.r-project.org/). The differentially expressed mRNA and lncRNAs were identified by the linear models for microarray analysis (Limma) method [12]. The DEGs were obtained with thresholds of ∣logFC∣ > 1.5 and *P* < 0.001. The hierarchical cluster analysis of differentially expressed mRNAs and lncRNAs was performed using CLUSTER 3.0 [13], and the hierarchical clustering heat map was visualized by Tree View [14].

### 2.2. Coexpression Network Construction and Analysis

In this study, as Hu et al. [15] described, the Pearson correlation coefficient of DEG-lncRNA pairs was calculated according to the expression value of them. The coexpressed DEG-lncRNA pairs with the absolute value of Pearson correlation coefficient ≥ 0.75 were selected, and the coexpression network was established by using cytoscape software. Cytoscape MCODE plug-in (version 3.4.0, available online: http://www.cytoscape.org/) was applied for visualization of the coexpression networks.

Gene coexpression analysis could be applied to related genes of unknown function with GO or to analysis candidate disease genes or to predict transcriptional regulatory mechanism [16].

### 2.3. GO and KEGG Pathway Analyses

To identify functions of DEGs in smoking-related lung cancer, we performed GO function enrichment analysis in 3 functional ontologies: biological process (BP), cellular component (CC), and molecular function (MF). KEGG pathway enrichment analysis was also performed to identify pathways enriched in smoking-related lung cancer using the DAVID system (https://david.ncifcrf.gov/). The *P* value less than 0.05 was considered significant.

### 2.4. Survival Analysis

GEPIA database (http://gepia.cancer-pku.cn/index.html) was used to predict the correlation between candidate gene expression and overall survival (OS) time or disease-free survival (DFS) time. The median expression of target was selected as cutoff to divide all TC samples as high and low groups. The probability of survival was estimated using the Kaplan-Meier method. The log-rank test was used to compare differences in survival times.

### 2.5. Statistical Analysis

The numerical data were presented as mean ± standard deviation (SD) of at least three determinations. Statistical comparisons between groups of normalized data were performed using *T*-test or Mann–Whitney *U* test according to the test condition. A *P* < 0.05 was considered statistical significance with a 95% confidence level.

## 3. Results

### 3.1. Identification of Differently Expressed lncRNAs in TC

**Figure 1:**
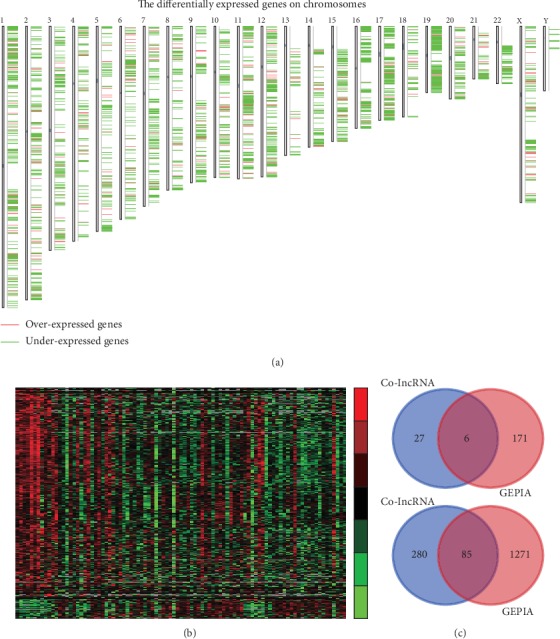
Identification of differently expressed lncRNAs in TC. (a) Chromosomal distribution of differently expressed genes in TC tissues using GEPIA database. (b) Hierarchical clustering analysis shows differential lncRNA expression between normal and TC samples by using Co-lncRNA database. (c, d) Venn diagrams display differently expressed lncRNAs in both databases.

GEPIA database was first analyzed. Our results identified 177 upregulated lncRNAs and 1359 downregulated lncRNAs in TC samples compared to normal tissues (Figure 1(a) and Supplementary Table 1). By analyzing Co-lncRNA database, 399 lncRNAs were found to be dysregulated in TC. Among these lncRNAs, 33 lncRNAs were overexpressed and 366 lncRNAs were suppressed in TC tissues compared to normal tissues (Figure 1(b)).

By performing integrated analysis of Co-lncRNA and GEPIA databases, a total of 6 lncRNAs were found to be upregulated and 85 lncRNAs were found to be downregulated in TC samples (Figure 1(c)). CATIP-AS1 is the most significantly downregulated lncRNA, and RP11-280O1.2 is the most significantly upregulated lncRNA in TC.

### 3.2. Downregulated lncRNAs Were Correlated to Longer Disease-Free Survival Time in TC

**Figure 2:**
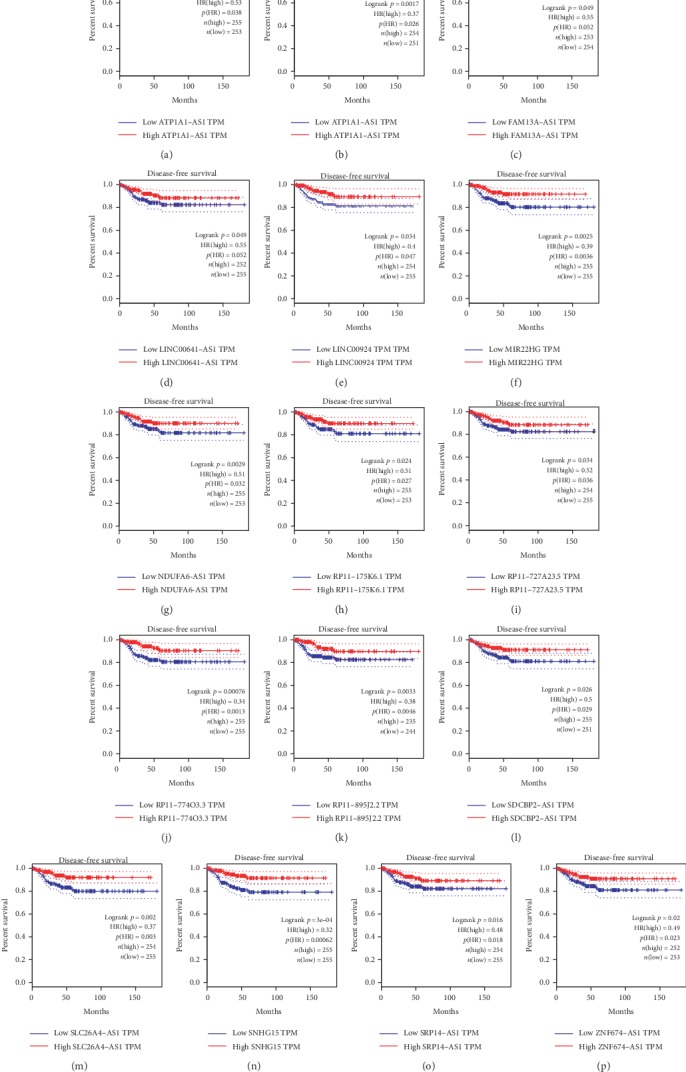
Identification of disease-free survival time related lncRNAs in TC. (a-p) Higher expression levels of ATP1A1-AS1 (a), CATIP-AS1 (b), FAM13A-AS1 (c), LINC00641 (d), LINC00924 (e), MIR22HG (f), NDUFA6-AS1 (g), RP11-175K6.1 (h), RP11-727A23.5 (i), RP11-774O3.3 (j), RP13-895J2.2 (k), SDCBP2-AS1 (l), SLC26A4-AS1 (m), SNHG15 (n), SRP14-AS1 (o), and ZNF674-AS1 (p) were significantly correlated to longer disease-free survival time in patients with TC.

Then, the GEPIA dataset was used to explore the correlation between lncRNA expression and overall survival time in TC. The median expression level of target gene was selected as the cutoff to divide all TC samples into high and low groups. Our analyses showed dysregulated lncRNAs were significantly correlated to the disease-free survival time in TC. Higher expression levels of ATP1A1-AS1, CATIP-AS1, FAM13A-AS1, LINC00641, LINC00924, MIR22HG, NDUFA6-AS1, RP11-175K6.1, RP11-727A23.5, RP11-774O3.3, RP13-895J2.2, SDCBP2-AS1, SLC26A4-AS1, SNHG15, SRP14-AS1, and ZNF674-AS1 were significantly correlated to longer disease-free survival time in patients with TC (Figures 2(a)-2(p)).

**Figure 3:**
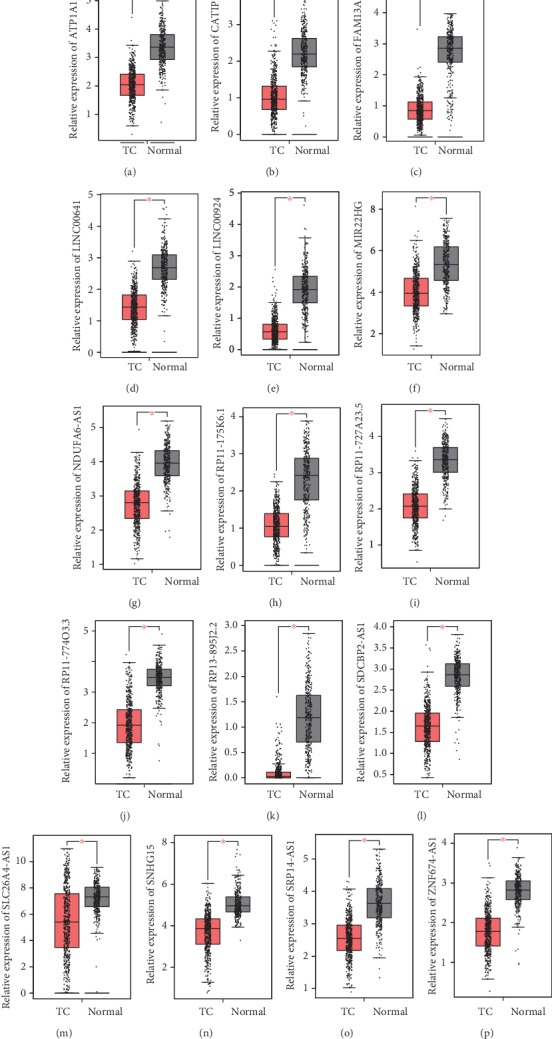
We identified downregulated lncRNAs in TC. (a-p) The expression levels of ATP1A1-AS1 (a), CATIP-AS1 (b), FAM13A-AS1 (c), LINC00641 (d), LINC00924 (e), MIR22HG (f), NDUFA6-AS1 (g), RP11-175K6.1 (h), RP11-727A23.5 (i), RP11-774O3.3 (j), RP13-895J2.2 (k), SDCBP2-AS1 (l), SLC26A4-AS1 (m), SNHG15 (n), SRP14-AS1 (o), and ZNF674-AS1 (p) were downregulated in TC samples compared to normal tissues.

Of note, we found that ATP1A1-AS1, CATIP-AS1, FAM13A-AS1, LINC00641, LINC00924, MIR22HG, NDUFA6-AS1, RP11-175K6.1, RP11-727A23.5, RP11-774O3.3, RP13-895J2.2, SDCBP2-AS1, SLC26A4-AS1, SNHG15, SRP14-AS1, and ZNF674-AS1 were significantly downregulated in TC samples compared to normal tissues (Figures 3(a)-3(p)). These results suggested these lncRNAs may serve as tumor suppressors in TC.

### 3.3. Construction of Differently Expressed lncRNAs Regulating Coexpression Network in TC

**Figure 4:**
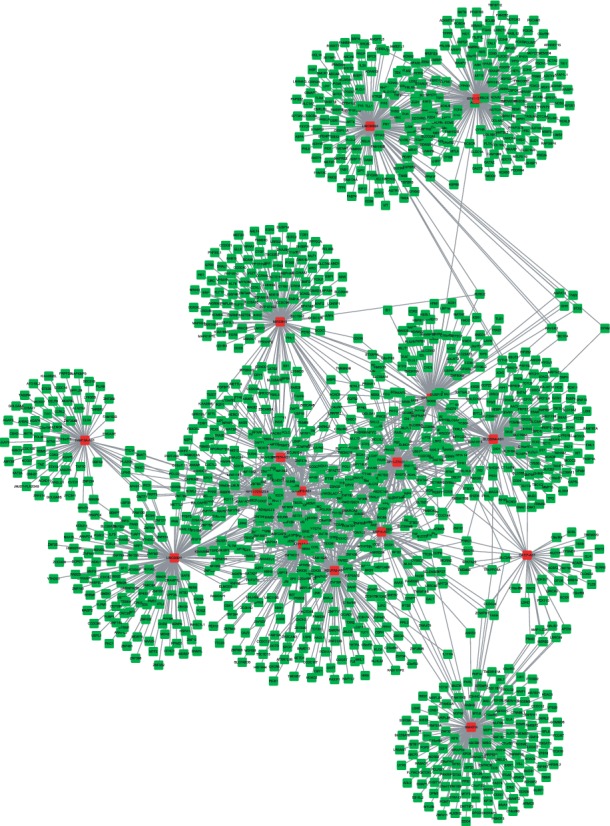
Coexpression network analysis of lncRNAs in TC. Coexpression network analysis of lncRNAs in TC. Red nodes: lncRNA; green nodes: mRNA.

Furthermore, we constructed differently expressed lncRNAs regulating coexpression network in TC. The Pearson correlation coefficients between lncRNA and mRNA was downloaded from GEPIA datasets. We selected the top 200 correlated genes as the potential targets of differently expressed lncRNAs. As shown in Figure 4, we found that this coexpression network contained 16 lncRNAs and 2698 mRNAs.

### 3.4. Bioinformatics Analysis of Differently Expressed lncRNAs in TC

**Figure 5:**
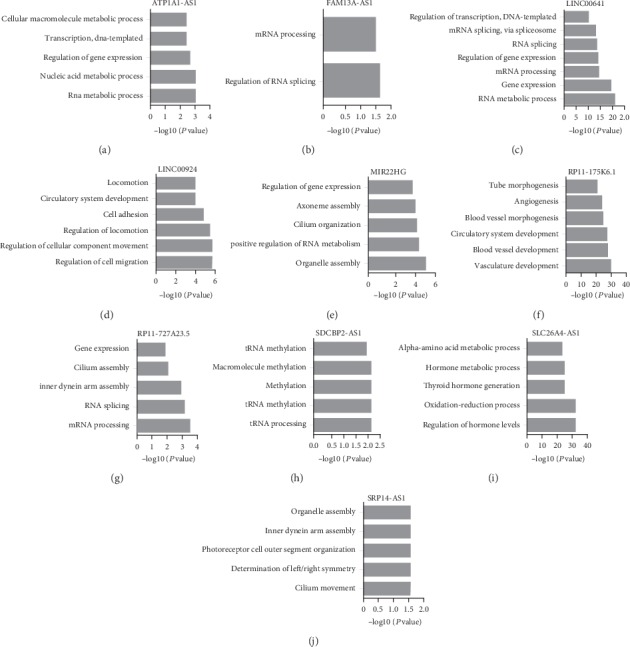
Bioinformatics analysis of differently expressed lncRNAs. (a-j) Bioinformatics analysis of ATP1A1-AS1 (a), FAM13A-AS1 (b), LINC00641 (c), LINC00924 (d), MIR22HG (e), RP11-175K6.1 (f), RP11-727A23.5 (g), SDCBP2-AS1 (h), SLC26A4-AS1 (i), and SRP14-AS1 (j) in TC.

Furthermore, we performed bioinformatics analysis for differentially expressed lncRNAs in TC (Figure 5). GO analysis showed that ATP1A1-AS1 [17] was involved in regulating the RNA metabolic process, the nucleic acid metabolic process, regulation of gene expression, transcription, DNA-templated, and cellular macromolecule metabolic process. FAM13A-AS1 [18] was involved in regulating RNA splicing and mRNA processing. LINC00641 was involved in regulating the RNA metabolic process; gene expression; mRNA processing; regulation of gene expression; RNA splicing; and mRNA splicing, via spliceosome, and transcription [19]. LINC00924 was associated with the regulation of cell migration, regulation of cellular component movement, regulation of locomotion, cell adhesion, circulatory system development, and locomotion. MIR22HG was involved in regulating organelle assembly, positive regulation of the RNA metabolic process, cilium organization, axoneme assembly, and regulation of gene expression [20]. RP11-175K6.1 was involved in regulating vasculature development, blood vessel development, circulatory system development, blood vessel morphogenesis, angiogenesis, and tube morphogenesis. RP11-727A23.5 was involved in regulating mRNA processing, RNA splicing, inner dynein arm assembly, cilium assembly, and gene expression. SDCBP2-AS1 was involved in regulating the tRNA process, tRNA methylation, methylation, macromolecule methylation, and tRNA modification [21]. SLC26A4-AS1 was involved in regulating regulation of hormone levels, the oxidation-reduction process, thyroid hormone generation, the hormone metabolic process, and the alpha-amino acid metabolic process [22]. SRP14-AS1 was involved in regulating cilium movement, determination of left/right symmetry, photoreceptor cell outer segment organization, inner dynein arm assembly, and organelle assembly (Figure 5(a)-5(j)).

## 4. Discussion

Thyroid cancer is a rare but a highly lethal form of thyroid cancer, which needs more attention. And lncRNAs had been demonstrated to play key roles in the progression of most human cancers, including thyroid cancer. For instance, DGCR5 played as a tumor suppressor in TC though binding to miR-2861 [23]. SNHG16 promoted TC proliferation and invasion through modulation of miR-497 [24]. GAS8-AS1 inhibited TC growth through miR-135b-5p/CCND2 axis. Of note, lncRNAs were also found to be dysregulated in TC, suggesting the potential prognostic value of lncRNAs. For example, a bioinformatics analysis study showed that FAM95B1 and UCA1 were correlated with cervical lymph node metastasis, tumor staging, and TC prognosis. Lu et al. reported that the dysregulation of RUNDC3A-AS1, FOXD-AS1, RUNDC3A-AS1 and FOXD-AS1 was correlated to a shorter overall survival time in patients with TC [25]. However, only a small part of lncRNAs were reported in TC. The expression pattern and molecular functions of most lncRNAs in TC remained unknown.

In our study, silico analyses were performed to identify TC-related important lncRNA. Co-lncRNA and GEPIA databases were used to identify differently expressed lncRNAs in TC. There are a total of 6 upregulated and 85 downregulated lncRNAs in TC samples compared to normal tissues. Among these lncRNAs, only few lncRNAs were reported in previous studies. For example, NR2F1-AS1 was found to be upregulated in TC samples. In hepatocellular carcinoma, knockdown of NR2F1-AS1 significantly suppressed cancer invasion, migration, and in vivo tumor growth [26]. Moreover, we for the first time identified 16 downregulated lncRNAs was correlated to a longer disease-free survival time in patients with TC, including ATP1A1-AS1, CATIP-AS1, FAM13A-AS1, LINC00641, LINC00924, MIR22HG, NDUFA6-AS1, RP11-175K6.1, RP11-727A23.5, RP11-774O3.3, RP13-895J2.2, SDCBP2-AS1, SLC26A4-AS1, SNHG15, SRP14-AS1, and ZNF674-AS1. The functions of these lncRNAs remained unclear. ATP1A1-AS1 is a novel lncRNA. A previous study showed ATP1A1-AS1 is a negative regulator of Na/K-ATPase *α*1 and involved in regulating cell proliferation in human kidney cells. LINC00641 was reported as a tumor suppressor in bladder cancer via sponging miR-197 [19]. SLC26A4-AS1was found to be associated with overall survival in gastric cancer. SNHG15 was reported to be downregulated in thyroid cancer and acted as a tumor suppressor in TC [27].

LncRNA coexpression network was widely used to explore the potential roles of novel lncRNAs in TC. For example, Zhang et al. revealed that HCG11 was involved in regulating the MAPK signaling pathway and gene transcription though coexpression analysis [28]. In this study, we constructed a network including 19 downregulated lncRNAs and 2698 mRNAs. Bioinformatics analysis showed these lncRNAs played crucial roles in TC progression. For example, ATP1A1-AS1, RP11-727A23.5, and LINC00641 were involved in regulating the RNA metabolic process. FAM13A-AS1 was involved in regulating RNA splicing. LINC00924 was associated with the regulation of cell migration and cell adhesion. MIR22HG was involved in regulating organelle assembly. RP11-175K6.1 was involved in regulating vasculature development. SDCBP2-AS1 was involved in regulating tRNA modification. SLC26A4-AS1 was involved in regulating hormone levels.

In conclusion, we identified 6 upregulated and 85 downregulated lncRNAs in TC samples. Moreover, we for the first time identified 16 downregulated lncRNAs was correlated to a longer disease-free survival time in patients with TC, including ATP1A1-AS1, CATIP-AS1, FAM13A-AS1, LINC00641, LINC00924, MIR22HG, NDUFA6-AS1, RP11-175K6.1, RP11-727A23.5, RP11-774O3.3, RP13-895J2.2, SDCBP2-AS1, SLC26A4-AS1, SNHG15, SRP14-AS1, and ZNF674-AS1. Bioinformatics analysis revealed these lncRNAs were involved in regulating the RNA metabolic process, cell migration, organelle assembly, tRNA modification, and hormone levels. This study will provide useful information to explore the potential candidate biomarkers for diagnosis, prognosis, and drug targets for TC.

## Data Availability

All the data in this manuscript can be accessed in Co-LncRNA (http://www.biobigdata.com/Co-LncRNA/).

## Abbreviations

TC: Thyroid cancer
Lnc-RNAs: Long noncoding RNAs
EMT: Epithelial-mesenchymal transition
RFS: Recurrence-free survival
GO: Gene Ontology
KEGG: Kyoto Encyclopedia of Genes and Genomes
BP: Biological process
CC: Cellular component
MF: Molecular function
OS: Overall survival
DFS: Disease-free survival.

## Supplementary Materials

Supplementary Materials Supplementary table 1: the differently expressed lncRNAs in TC by using GEPIA database.
